# Chemoenzymatic approaches to plant natural product inspired compounds

**DOI:** 10.1039/d2np00008c

**Published:** 2022-03-28

**Authors:** Rebecca Roddan, Eve M. Carter, Benjamin Thair, Helen C. Hailes

**Affiliations:** Department of Chemistry, University College London Christopher Ingold Building London WC1H 0AJ UK h.c.hailes@ucl.ac.uk

## Abstract

Covering: 2003 up to the end of 2021

Complex molecules produced by plants have provided us with a range of medicines, flavour and fragrance compounds and pesticides. However, there are challenges associated with accessing these in an economically viable manner, including low natural abundance and the requirement for complex multi-step synthetic strategies. Chemoenzymatic approaches provide a valuable alternative strategy by combining traditional synthetic methods with biocatalysis. This review highlights recent chemoenzymatic syntheses towards plant natural products and analogues, focusing on the advantages of incorporating biocatalysts into a synthetic strategy.

## Abstract 2

Chemoenzymatic routes to plant natural products and analogues are described, highlighting the advantages of incorporating biocatalysis into synthetic strategies.

## Graphical abstract

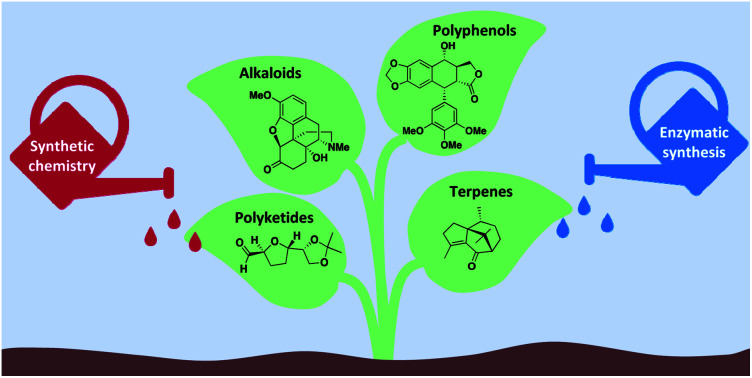

## 1 Introduction

The biological effects of natural products (NPs) have been exploited by humans for millennia. Many are economically valuable, with uses as pigments, medicines, insecticides, and food additives.^1^ In particular, plant secondary metabolites were among the first recognised medicines (*e.g.* morphine in 1827) and many are widely known (*e.g.* aspirin, quinine and caffeine).^2^ However, the diversity of complex scaffolds in these compounds creates obstacles for their traditional synthesis at scale.

In plants, secondary metabolites are synthesized by elaborate, enzyme-catalyzed pathways. Once a plant extract is found to possess useful properties, isolating and identifying the active components can be incredibly challenging due to their low natural abundance and the presence of other, structurally similar molecules. Purification can therefore be laborious and result in poor isolated yields.^3^ Moreover, structural determination, even by well-established spectroscopic methods, can be problematic. Indeed, it is still the case that structures of NPs are revised in the literature, such as the antiproliferative nagilactone I.^4^

Traditional synthetic routes to NPs have the potential to improve yields, avoid difficult purifications and offer structural certainty. Bypassing these challenges is crucial for increasing the number of compounds available for high-throughput screening in the discovery of novel therapeutics. The total synthesis of complex NPs from readily available precursors has led to some impressive and commercially viable pathways.^5^ However, the structural complexity of NPs makes cost-effective, chemo-, regio- and stereoselective syntheses often unattainable.

Fermentation routes which exploit microbial products have been used since the Neolithic age to generate foods and beverages. More recently, advances in synthetic biology and increasing access to genetic sequencing data have made it possible to produce non-microbial NPs by similar methods.^6^ Notable examples include the generation of the plant NPs noscapine,^7^ and hyoscyamine and scopolamine^8^ in yeast. However, significant biological engineering efforts are required for the production of an individual compound. The use of recombinantly expressed enzymes to perform reactions *in vitro* with high selectivity under benign reaction conditions, known as biocatalysis, is another method by which NPs and analogues can be produced. However, issues of enzyme reusability, stability and limited substrate scope can limit more widespread usage.^9^

Chemoenzymatic cascade approaches harness the selectivity of biocatalysis and the versatility of traditional synthetic methods. Their combination in sequence, or together in one-pot reactions, can avoid the downfalls of each individual strategy. Benefits include the telescoping of unstable intermediates and the creation of branch points for the synthesis of analogues for drug discovery purposes.^10^ In this review, we describe notable examples of chemoenzymatic cascades towards plant-inspired NPs, categorised by the common building blocks of each NP type.

## 2 Alkaloids

**Scheme 1:**
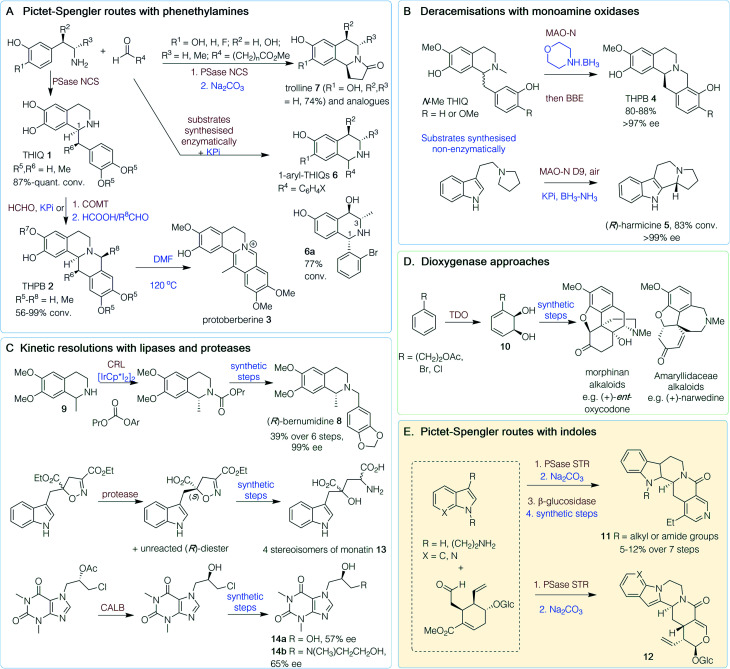
Chemoenzymatic routes to plant alkaloids. PSase = Pictet–Spenglerase, NCS = norcoclaurine synthase, COMT = catechol-*O*-methyltransferase, BBE = berberine bridge enzyme, MAO-N = monoamine oxidase variant, CALB = *Candida antarctica* lipase B, CRL = *Candida rugosa* lipase, TDO = toluene dioxygenase, STR = strictosidine synthase. Red/brown text indicates enzymatic step, blue text indicates synthetic step.

Alkaloids are a structurally diverse family of nitrogen-containing compounds biosynthesised, in most cases, from amino acids. Some of the most historically important medicines are alkaloids, with widely known examples including morphine, caffeine, cocaine and nicotine.^11^ Here, selected chemoenzymatic routes are highlighted by structural type and in Scheme 1 by the enzyme strategy adopted.

The heterocyclic isoquinoline (IQ) scaffold is commonly found in plant-derived alkaloids. Chemoenzymatic routes to natural and non-natural IQs have exploited the wide substrate scope and high stereoselectivity of the Pictet–Spenglerase (PSase) norcoclaurine synthase (NCS) (Scheme 1A).^12^ A one-pot route to tetrahydroprotoberberine (THPB) alkaloids from dopamine was achieved *via* a ‘triangular cascade’, involving first the *in situ* generation of the corresponding aldehyde (R^4^ = CH_2_C_6_H_4_(OH)_2_) using a transaminase from *Chromobacterium violaceum* (*Cv*TAm). Reaction of the amine and aldehyde components catalyzed by *Thalictrum flavum* NCS (*Tf*NCS) generated tetrahydroisoquinoline (THIQ) 1 (*S*)-norlaudanosoline (R^5^, R^6^ = H) in an 87% conversion and >99% enantiomeric excess (ee) at C-1 (Scheme 1A). Subsequent addition of formaldehyde triggered another Pictet–Spengler (PS) reaction, catalyzed by potassium phosphate (KPi), to give the THPB 2 (R^5^–R^8^ = H). Reactions were performed on a 0.5 mmol scale giving a 42% isolated yield (56% conversion) and high stereoselectivity (>95% ee *S*-isomer).^13^ Further cascades were developed towards 13-methyl-THPBs, similar to those isolated from *Corydalis* plants. These routes exploited the ability of a *Tf*NCS variant (M97V) to perform a kinetic resolution with α-methyl substituted aldehydes in quantitative conversions (R^4^ = CHMeC_6_H_4_(OMe)_2_), forming two well-defined chiral centres in THIQ 1 (R^5^, R^6^ = Me), with (*S*)-stereochemistry at C-1 again. This reaction, in conjunction with regioselective catechol-*O*-methyltransferases (COMT) and chemical PS reactions with formaldehyde and acetaldehyde, gave a range of THPBs 2 (R^5^, R^6^ = Me; R^7^, R^8^ = H or Me) with conversions of 56–99% and good stereoselectivities, and protoberberine 3 after heating in DMF.^14^

An alternative route to THPBs 4 has been reported using the berberine bridge enzyme (BBE), which natively performs an enantioselective C–C bond forming reaction (Scheme 1B). To enable complete conversion of racemic *N*-Me THIQs, an (*R*)-selective monoamine oxidase variant (MAO-N) was also used in an initial deracemisation step with morpholine BH_3_. Subsequent addition of the BBE gave (*S*)-THPBs 4 (R = H or OMe) in 80–88% yield and >97% ee, and the one-pot cascade could be performed on a 150 mg scale.^15^ A related approach has been used to generate (*R*)-harmicine 5, an indole alkaloid, with MAO-N for deracemization in tandem with a racemic PS reaction in overall 83% conversions in one-pot (Scheme 1B).^16^

Starting from 3-hydroxybenzaldehyde and pyruvate, single-isomer, trisubstituted 1,3,4-THIQs have been generated using cascades with a carboligase (*Ec*AHAS-I), transaminase (*Cv*TAm) and a stereoselective PS reaction using either NCS or KPi, and phenylacetaldehyde or *o*-bromobenzaldehyde, respectively, to give opposing C-1 stereochemistries. The stereoselectivity of the KPi-mediated PS reaction to the 1-aryl-THIQ 6 (Scheme 1A) was influenced by the phenylethylamine stereochemistries at C-3 and C-4 to give the preferred epimer 6a in a 77% conversion over 3 steps.^17^ An alternative one-pot route to 6 has also been developed, using a laccase/TEMPO-mediated oxidation to generate benzaldehydes from the corresponding benzyl alcohols. The reaction was performed with *meta*-tyramine in KPi to facilitate a regioselective PS reaction and racemic THIQs 6 (R^1^–R^3^ = H, R^4^ = C_6_H_4_X) were generated in yields of 32–87% (Scheme 1A).^18^ Trolline, an alkaloid with antiviral and antibacterial properties, could also be formed in a one-pot reaction from phenethylamines and a linear aldehyde with a terminal ester. An NCS-mediated reaction, followed by lactam formation under mildly basic conditions, resulted in (*S*)-trolline formation (7, R^1^ = OH, R^2^ = R^3^ = H) in 74% isolated yield and >95% ee, and several analogues were generated.^19^

The THIQ (*R*)-bernumidine 8 has been generated using a chemoenzymatic dynamic kinetic resolution. Starting from synthesised (*rac*)-salsolidine 9, an iridium-based catalyst for amine racemization combined with *Candida rugosa* lipase (CRL) generated (*R*)-salsolinol propyl carbamate (Scheme 1C) in 68% yield and 99% ee. Hydrolysis of the carbamate gave (*R*)-9 and subsequent chemical transformations generated (*R*)-8, in an overall six-step route from (*rac*)-9, in 39% yield and 99% ee.^20^

Morphine and analogues are attractive synthetic targets, however, the complexity of the molecular scaffold has made cost-effective, scalable syntheses somewhat elusive. Significant efforts towards these compounds by the Hudlicky group over the past 25 years have involved a chemoenzymatic approach. The first key step was a toluene dioxygenase (TDO)-mediated dihydroxylation (whole-cell fermentation) of substituted benzenes to give enantiopure *cis*-dihydrocatechols 10, thus incorporating the key stereochemistry required into the C-ring of morphinan alkaloids (Scheme 1D).^21,22^ A 2015 review has highlighted how the subsequent chemical steps have been developed over the years.^23^ This approach has also been used to generate other families of alkaloids by the Hudlicky, Banwell and Willis groups, such as those isolated from plants of the *Amaryllidaceae* genus (Scheme 1D).^24–29^

Another PSase, strictosidine synthase (STR), is involved in the biosynthesis of indole alkaloids from the *Apocynaceae* plant family and has likewise found use in chemoenzymatic syntheses.^12,30^ Examples include the *in vitro* synthesis of *N*-substituted tetrahydroangustines, where STR coupled the natural substrates tryptamine and secologanin in the first step (Scheme 1E), followed by a base-catalysed intramolecular lactamisation.^31^ Reduction, β-glucosidase-mediated cleavage of the glycosidic bond and further chemical steps gave *N*-substituted products, 11, in 5–12% yield over 7 steps. Beyond the natural substrate scope, Wu *et al.*^32^ used a range of *N*-substituted indole derivatives in an STR-mediated reaction with secologanin as a first step in the chemoenzymatic synthesis of piperazino-indole alkaloids. Intramolecular lactamisation gave the pentacyclic alkaloids 12. Another indole alkaloid, monatin 13, has been synthesized using proteases from *Aspergillus oryzae* to resolve a key diester intermediate to a single acid enantiomer (>97% ee for the remaining diester).^33^ Subsequent non-enzymatic steps and separation of the diastereoisomers gave the four stereoisomers of 13 (Scheme 1C).

In the chemoenzymatic synthesis of two xanthine-containing alkaloids (Scheme 1C), a lipase-mediated kinetic resolution was utilised. Here, the widely used, *Candida antarctica* lipase B (CALB) immobilized on an acrylic resin was used which is tolerant to organic solvents.^34^ This gave a key chlorohydrin intermediate in 38% yield and 71% ee on a 500 mg scale. Further chemical transformations gave 14a and 14b, (*R*)-diprophylline and (*S*)-xanthinol nicotinate, in 57% and 65% ee, respectively.^35^

## 3 Terpenoids

**Scheme 2:**
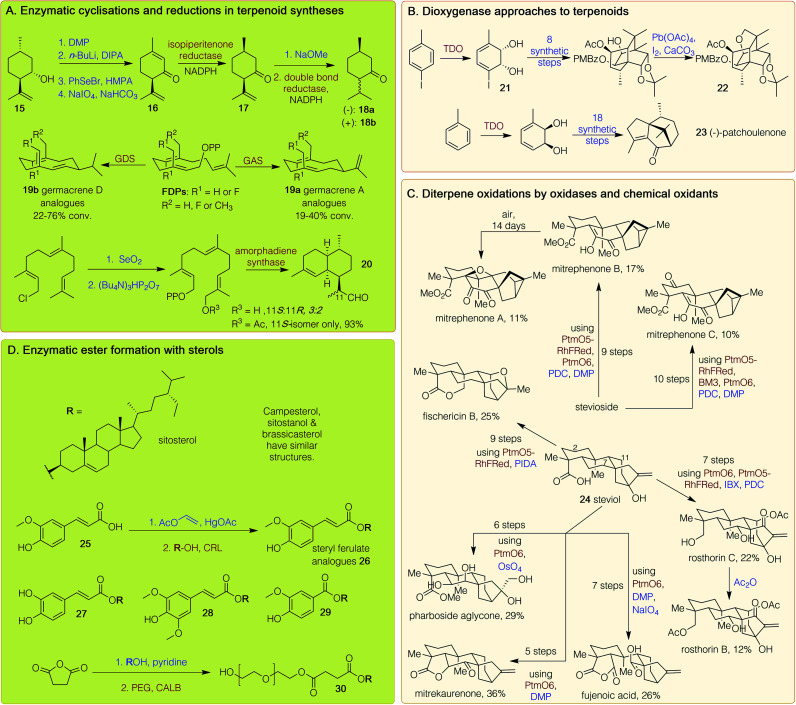
Chemoenzymatic routes to terpenoids and steroids. GDS = germacrene D synthase, GAS = germacrene A synthase, TDO = toluene dioxygenase, CALB = *Candida antarctica* lipase B, CRL = *Candida rugosa* lipase. DIPA = diisopropylamine, DMP = Dess–Martin periodinane, IBX = 2-iodoxybenzoic acid, NADPH = nicotinamide adenine dinucleotide phosphate, PDC = pyridinium dichromate, PEG = polyethylene glycol, PIDA = (diacetoxyiodo)benzene. Red/brown text indicates enzymatic step, blue text indicates synthetic step.

Terpenoids, natural products with repeating C_5_ isoprene units as building blocks, are produced predominantly by plants. Biosynthetically, after addition of the C_5_ units in a head-to-tail fashion, most terpenoids are cyclised and then further modified. They have numerous applications as pharmaceuticals and fragrance compounds as well as biological relevance such as squalene, the precursor to steroids. Many monoterpenoids, comprised of C_10_ units, have fragrant odours and are used in the perfume and insecticide industries. The most commonly used monoterpenoids, such as menthol, camphor and limonene, are readily isolated from natural sources. However, the chemoenzymatic syntheses of all intermediates in the peppermint pathway, and of menthone and isomenthone, have been reported. Initially, (+)-isopulegol 15 was oxidised to the corresponding ketone, then selenylated, further oxidised and eliminated, giving (−)-isopiperitenone 16 (Scheme 2A).^36^ A preparative scale biotransformation with isopiperitenone reductase readily gave (+)-*cis*-isopulegone 17 on a ∼600 mg scale. This was isomerised and reduced using a double bond reductase from *Nicotiana tabacum* to produce (−)-menthone 18a and (+)-isomenthone 18b in 37% and 31% yield respectively, which could be separated by column chromatography.^36^

Terpene synthases have significant potential in chemoenzymatic routes to terpenoids. An example includes synthesis of the macrocyclic sesquiterpenes (C_15_) germacrene A 19a and germacrene D 19b, along with fluorinated and methylated analogues. Farnesyl diphosphate (FDP) and analogues were synthesised and then reacted with germacrene A synthase (GAS) and germacrene D synthase (GDS) from *Solidago canadensis* to provide the products 19a and 19b respectively, with higher yields observed for 19b analogues (Scheme 2A).^37^ The sesquiterpenoid endoperoxide artemisinin is widely used as a first-line treatment for malaria. There are several chemical and enzymatic syntheses reported but worldwide supply predominantly relies on extraction from the plant *Artemisia annua* due to the high costs of these processes. A shorter chemoenzymatic route has been published to an artemisinin intermediate involving the initial selenium dioxide-mediated oxidation of commercially available (*E*,*E*)-farnesyl chloride, followed by diphosphorylation of the resulting chloride. Amorphadiene synthase converted this into dihydroartemisinic aldehyde 20 as a 3 : 2 mixture of stereoisomers (Scheme 2A). Selectivity was improved when the primary alcohol was acetylated; treatment of this with amorphadiene synthase provided 20 as a single isomer in 93% yield.^38^

Further routes to sequiterpenoids have again utilised toluene dioxygenase (TDO) (Scheme 2B) to establish the stereochemical handles. Tashironins, isolated from species of the genus *Illicium*, have complex, highly oxygenated polycyclic structures and their reported biological properties include action against hepatitis B virus.^39^ A chemoenzymatic route towards these first reacted TDO with *p*-iodotoluene to give 21. This was converted to a polycyclic alcohol in four steps, then acetylated, diastereoselectively *cis*-dihydroxylated and converted into the corresponding *p*-methoxyphenylbenzylidene acetal, which was oxidatively cleaved to provide a *p*-methoxybenzoate. An intramolecular alkoxy radical-mediated cyclisation was then triggered upon exposure to lead tetraacetate and iodine under ultrasonic irradiation, providing the key intermediate 22 with a yield of 90% for further modification to the tashironins.^39^ The same authors again employed TDO but instead, starting with toluene to provide *cis*-1,2-dihydrocatechol which was used in two distinct syntheses of the sesquiterpene (−)-patchoulenone 23, which has been shown to possess anti-malarial and anti-fungal properties.^40^

A significant transformation in the synthesis of terpenes is the selective enzymatic oxidation of a specific carbon on a complex scaffold, and the characterisation of these enzymes provides a toolkit for use in syntheses. The Renata group have used a chemoenzymatic approach to access nine complex diterpenoid (C_20_) NPs from stevioside or the aglycone *ent*-steviol 24 (Scheme 2C). A P450 monooxygenase, PtmO5, from the platensimycin biosynthetic pathway, catalysed a remote C–H hydroxylation at the C-11 position; its fusion with the reductase domain of P450_RhF_ gave PtmO5-RhFRed. The α-ketoglutarate-dependant dioxygenase PtmO6 from the same pathway was found to hydroxylate at C-7, while a variant of P450_BM3_ selectively hydroxylated the C-2 position.^41^ These three enzymes were combined with a range of chemical oxidants (such as Dess–Martin periodinane (DMP), osmium tetroxide, 2-iodoxybenzoic acid (IBX) (diacetoxyiodo)benzene (PIDA) and pyridinium dichromate (PDC)) to provide nine highly oxidised terpenoids in 10 steps or fewer, all in respectable yields (Scheme 2C).^41^

Phytosterols are plant steroids typically biosynthesised from lanosterol, and their derivatives occur naturally in vegetable oils, fruits, and cereal grains. They have a fused polycyclic structure and occur as both free alcohols and conjugated esters. The esters are rapidly hydrolysed by intestinal enzymes, producing the physiologically active sterols. Phytosteryl ferulates have antioxidant, serum cholesterol-lowering, anti-inflammatory, and antitumor properties, and have been synthesised in a chemoenzymatic route (Scheme 2D). First, ferulic acid 25 was reacted with vinyl acetate and a mercury acetate catalyst to give vinyl ferulate. This underwent esterification with a range of phytosterols using CRL, forming several steryl ferulates 26 such as sitosteryl ferulate, sitostanyl ferulate, and campesteryl ferulate in approximately 90% yield. These ferulates possessed higher antioxidant activity than ferulic acid and were thus suggested as alternative food antioxidants.^42^ Phytosteryl caffeates 27,^43^ sinapates 28 ^44^ and vanillates 29 ^44^ were synthesised by the same route from the respective acids. A hydrophilic phytosteryl ester was also synthesised *via* the esterification of phytosterols with succinic anhydride, followed by a CALB-catalysed esterification with polyethylene glycol to give 30. By increasing the hydrophilicity of phytosteryl esters, they could be more readily incorporated into high water-content food products.^45^

## 4 Polyketides

Polyketides are a diverse group of natural products formed by polyketide synthase complexes. Many are medicinally relevant, including the tetracycline antibiotic doxycycline and macrolide polyketide erythromycin,^46^ with the majority of medicinally relevant NP examples from bacterial sources. Microbial systems have however been engineered for the production of plant polyketides using type III plant polyketide synthase (PKS) pathways.^47^

**Scheme 3:**
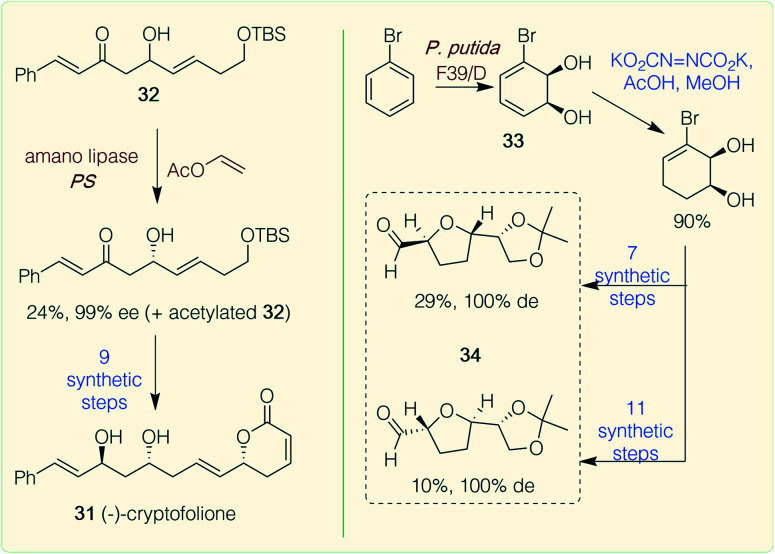
Chemoenzymatic routes to polyketides. Red/brown text indicates enzymatic step, blue text indicates synthetic step.

A few examples of chemoenzymatic routes to plant polyketides have been reported. Examples include the synthesis of cryptofolione 31 (Scheme 3), a δ-lactone found in stem bark of two *Cryptocarya* plants. A recent chemoenzymatic route by Vaithegi *et al.*^48^ used amano lipase PS in the presence of vinyl acetate to resolve one of the chiral centres in a key intermediate 32 (99% ee); 9 subsequent chemical steps gave (−)-cryptofolione 31.^48^ Another example is the acetogenins which are characterized by *trans*-tetrahydrofuran (THF) rings joined to α-hydroxylated carbon chains. Over 400 examples have been found in tropical plants from the *Annonaceae* family, many of which have potent biological activities. Work by Ramos *et al.* described the biocatalytic dihydroxylation of bromobenzene again using a dioxygenase (*Pseudomonas putida* F39/D whole cell system) to generate the key starting material 33. A regioselective reduction of a diimide formed *in situ* was high yielding and subsequent chemical steps afforded a range of ‘acetogenin-like’ *trans*-THF cores 34, in high steroselectivities.^49^

## 5 Polyphenols

**Scheme 4:**
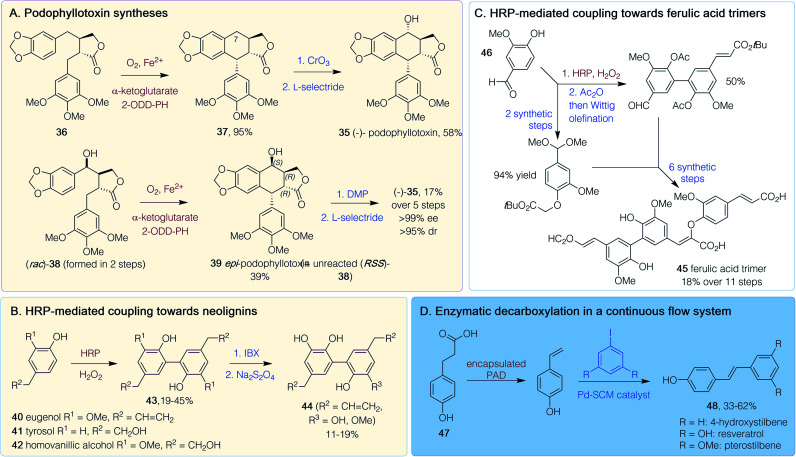
Polyphenol chemoenzymatic syntheses. 2-ODD-PH = 2-oxoglutarate-dependant dioxygenase, DMP = Dess–Martin periodinane, HRP = Horse radish peroxidase, IBX = 2-iodoxybenzoic acid, PAD = phenolic acid decarboxylase. Red/brown text indicates enzymatic step, blue text indicates synthetic step.

Polyphenols are characterized by repeating phenolic units and biosynthetically arise through phenylpropanoid and plant PKS pathways. They typically have molecular weights of between 500–4000 Da and are often highly conjugated so have applications as dyes. Due to their propensity for oxidation, they can act as plant antioxidants, which has led to interest in their medicinal potential.^50^ Despite there being relatively few polyphenol pharmaceuticals, several chemoenzymatic routes to plant inspired compounds have been developed.

(−)-Podophyllotoxin 35 is a potent microtubule depolymerization agent and a topical antiviral, while analogues such as etoposide and teniposide are effective in cancer treatments so there is interest in generating further analogues. Two chemoenzymatic routes to (−)-35 were published in 2019 (Scheme 4A).^51,52^ One was *via* the chemoselective synthesis of a single diastereomer of intermediate 36 using an Evans' oxazolidinone approach.^51^ The subsequent key enzymatic step utilized 2-oxoglutarate-dependent dioxygenase (2-ODD-PH) from the podophyllotoxin biosynthetic pathway. Impressive yields (95%) of 37 were achieved on a gram scale after co-expression of 2-ODD-PH with chaperones GroES and GroEL to improve enzyme solubility. Chemical oxidation at C-7, followed by a reduction, gave (−) 35 in 58% yield. Various methylated and cyclic acetal substitutions on the phenyl rings were tolerated to give analogues.^51^ Another route also used 2-ODD-PH, to provide a stereoselective C–C-bond formation.^52^ Here, the precursor (*rac*)-38 was generated in 2 steps and reaction with 2-ODD-PH lead to a kinetic resolution giving *epi*-podophyllotoxin 39 (39% yield at 2 g scale), leaving unreacted 38. Issues with enzyme insolubility were improved by a late induction. The enzyme was shown to be non-stereoselective for the hydroxylation unless the relative stereoconfiguration was that of 38. Product 39 was converted into (−)-35 in two further non-enzymatic steps (Scheme 4A), giving an overall yield for the synthesis of (−)-35 of 17% over five steps.^52^

Chemoenzymatic routes have also been developed towards dimeric neolignins inspired by magnolol with potent yeast α-glucoside activity: a property useful for finding new antidiabetic therapies.^53^ Starting from eugenol 40, tyrosol 41 or homovanillic acid 42, horseradish peroxidase (HRP)-mediated oxidative coupling (19–45% yield) gave 43 (Scheme 4B). Use of a 2-iodoxybenzoic acid (IBX)-mediated *ortho*-demethylation with 43 (R^2^ = CH=CH_2_) gave dimeric neolignans 44. Such C–C bond forming reactions with HRP have also been exploited in the synthesis of dehydrotrimers 45, of ferulic acid, starting from vanillin 46 (Scheme 4C). This route was performed in 10 steps, with an 18% overall yield involving HRP-mediated aryl–aryl coupling as the key initial step, followed by Wittig olefinations, an aldol-like condensation and an orthogonal protecting group strategy.^54^ Resveratrol, an antioxidant stilbenoid, has promising anticancer properties, although a lack of bioavailability and a poor side effect profile has hindered clinical usage. A chemoenzymatic tandem route has been developed in continuous flow starting from coumaric acid 47 (Scheme 4D). Decarboxylation by encapsulated *B. subtilis* phenolic acid decarboxylase (PAD) was followed by a Heck coupling of the resultant vinylphenol to an aryl iodide. Resveratrol and methoxy- and dehydroxylated analogues 48 could be generated in 33–62% yield on a 1 mol scale in just 1 h.^55^

## 6 Conclusions

This highlight summarises key chemoenzymatic syntheses for plant natural product inspired compounds. Many strategies differ drastically from the biosynthetic routes, emphasising the advantages of applying both chemical and biocatalytic expertise. The exploitation of chemo- and stereoselectivities exhibited by biocatalysts has allowed for highly selective reactions to be performed on much larger scales than in nature, making these more viable industrial options. The use of enzymes to install stereochemistries also enables a greener alternative to expensive and toxic metal catalyst routes that are widely used in industry. Advances in genetic sequencing, biocatalyst availability and flow technologies may soon allow more widespread adoption of chemoenzymatic reactions for natural product synthesis.

## 7 Author contributions

The manuscript was written by all authors, with the order reflecting the contributions by R. R., E. M. C., and B. T. All authors have given approval to the final version of the manuscript.

## 8 Conflicts of interest

There are no conflicts to declare.

## Supplementary Material

## References

[cit1] Newman D. J., Cragg G. M. (2020). J. Nat. Prod..

[cit2] Norn S., Kruse P., Kruse E. (2005). Dan Med. Arbog..

[cit3] Bucar F., Wube A., Schmid M. (2013). Nat. Prod. Rep..

[cit4] Chhetri B. K., Lavoie S., Sweeney-Jones A. M., Kubanek J. (2018). Nat. Prod. Rep..

[cit5] Baran P. S. (2018). J. Am. Chem. Soc..

[cit6] Pham J. V., Yilma M. A., Feliz A., Majid M. T., Maffetone N., Walker J. R., Kim E., Cho H. J., Reynolds J. M., Song M. C., Park S. R., Yoon Y. J. (2019). Front. Microb..

[cit7] Li Y., Li S., Thodey K., Trenchard I., Cravens A., Smolke C. D. (2018). PNAS.

[cit8] Srinivasan P., Smolke C. D. (2020). Nature.

[cit9] Truppo M. D. (2017). ACS Med. Chem. Lett..

[cit10] García-Junceda E., Lavandera I., Rother D., Schrittwieser J. H. (2015). J. Mol. Catal. B: Enzym..

[cit11] Heinrich M., Mah J., Amirkia V. (2021). Molecules.

[cit12] Roddan R., Ward J. M., Keep N. H., Hailes H. C. (2020). Curr. Opin. Chem. Biol..

[cit13] Lichman B. R., Lamming E. D., Pesnot T., Smith J. M., Hailes H. C., Ward J. M. (2015). Green Chem..

[cit14] Roddan R., Subrizi F., Broomfield J., Ward J. M., Keep N. H., Hailes H. C. (2021). Org. Lett..

[cit15] Schrittwieser J. H., Groenendaal B., Resch V., Ghislieri D., Wallner S., Fischereder E.-M., Fuchs E., Grischek B., Sattler J. H., Macheroux P., Turner N. J., Kroutil W. (2014). Angew. Chem., Int. Ed..

[cit16] Ghislieri D., Green A. P., Pontini M., Willies S. C., Rowles I., Frank A., Grogan G., Turner N. J. (2013). J. Am. Chem. Soc..

[cit17] Erdmann V., Lichman B. R., Zhao J., Simon R. C., Kroutil W., Ward J. M., Hailes H. C., Rother D. (2017). Angew. Chem., Int. Ed..

[cit18] Klein A. S., Albrecht A. C., Pietruszka J. (2021). Catalysts.

[cit19] Zhao J., Lichman B. R., Ward J. M., Hailes H. C. (2018). Chem. Commun..

[cit20] Corrêa B. K., Silva T. R. C., Raminelli C. (2018). Tetrahedron Lett..

[cit21] Makarova M., Endoma-Arias M. A. A., Dela Paz H. E., Simionescu R., Hudlicky T. (2019). J. Am. Chem. Soc..

[cit22] Endoma-Arias M. A., Dela Paz H., Hudlicky T. (2019). Molecules.

[cit23] Reed J. W., Hudlicky T. (2015). Acc. Chem. Res..

[cit24] Endoma-Arias M. A. A., Hudlicky T. (2016). Chem.–Eur. J..

[cit25] Matveenko M., Banwell M. G., Willis A. C. (2008). Tetrahedron.

[cit26] Schwartz B. D., Banwell M. G., Cade I. A. (2011). Tetrahedron Lett..

[cit27] White L. V., Schwartz B. D., Banwell M. G., Willis A. C. (2011). J. Org. Chem..

[cit28] Banwell M. G., Ma X., Karunaratne O. P., Willis A. C. (2010). Aust. J. Chem..

[cit29] Kokas O. J., Banwell M. G., Willis A. C. (2007). Tetrahedron.

[cit30] Zhu H., Kerčmar P., Wu F., Rajendran C., Sun L., Wang M., Stöckigt J. (2015). Curr. Med. Chem..

[cit31] Cai Y., Zhu H., Alperstein Z., Yu W., Cherkasov A., Zou H. (2017). ACS Chem. Biol..

[cit32] Wu F., Zhu H., Sun L., Rajendran C., Wang M., Ren X., Panjikar S., Cherkasov A., Zou H., Stöckigt J. (2012). J. Am. Chem. Soc..

[cit33] Bassoli A., Borgonovo G., Busnelli G., Morini G., Drew M. G. B. (2005). Eur. J. Org. Chem..

[cit34] Ortiz C., Ferreira M. L., Barbosa O., Dos Santos J. C. S., Rodrigues R. C., Berenguer-Murcia Á., Briand L. E., Fernandez-Lafuente R. (2019). Catal. Sci. Technol..

[cit35] Borowiecki P., Młynek M., Dranka M. (2021). Bioorg. Chem..

[cit36] Cheallaigh A. N., Mansell D. J., Toogood H. S., Tait S., Lygidakis A., Scrutton N. S., Gardiner J. M. (2018). J. Nat. Prod..

[cit37] Cascón O., Touchet S., Miller D. J., Gonzalez V., Faraldos J. A., Allemann R. K. (2012). Chem. Commun..

[cit38] Demiray M., Tang X., Wirth T., Faraldos J. A., Allemann R. K. (2017). Angew. Chem., Int. Ed..

[cit39] Sharma M. K., Banwell M. G., Willis A. C. (2015). J. Org. Chem..

[cit40] Banwell M. G., Hockless D. C. R., McLeod M. D. (2003). New J. Chem..

[cit41] Zhang X., King-Smith E., Dong L. B., Yang L. C., Rudolf J. D., Shen B., Renata H. (2020). Science.

[cit42] Tan Z., Shahidi F. (2011). J. Agric. Food Chem..

[cit43] Tan Z., Shahidi F. (2012). Food Chem..

[cit44] Tan Z., Shahidi F. (2013). Food Chem..

[cit45] He W. S., Hu D., Wang Y., Chen X. Y., Jia C. S., Ma H. L., Feng B. (2016). Food Chem..

[cit46] Hutchings M. I., Truman A. W., Wilkinson B. (2019). Curr. Opin. Microbiol..

[cit47] Lussier F. X., Colatriano D., Wiltshire Z., Page J. E., Martin V. J. J. (2012). Comput. Struct. Biotechnol. J..

[cit48] Vaithegi K., Prasad K. R. (2021). Tetrahedron.

[cit49] Ramos J. C., Brovetto M., Seoane G. A. (2013). Org. Lett..

[cit50] Pandey K. B., Rizvi S. I. (2009). Oxid. Med. Cell. Longevity.

[cit51] Li J., Zhang X., Renata H. (2019). Angew. Chem., Int. Ed..

[cit52] Lazzarotto M., Hammerer L., Hetmann M., Borg A., Schmermund L., Steiner L., Hartmann P., Belaj F., Kroutil W., Gruber K., Fuchs M. (2019). Angew. Chem., Int. Ed..

[cit53] Pulvirenti L., Muccilli V., Cardullo N., Spatafora C., Tringali C. (2017). J. Nat. Prod..

[cit54] Mouterde L. M. M., Flourat A. L., Cannet M. M. M., Ducrot P. H., Allais F. (2013). Eur. J. Org. Chem..

[cit55] Lackner F., Hiebler K., Grabner B., Gruber-Woelfler H. (2020). Catalysts.

